# Evaluation of a pharmacist-led intervention to improve medication adherence in patients initiating dabigatran treatment: a comparison with standard pharmacy practice in Poland

**DOI:** 10.1186/s12875-022-01821-9

**Published:** 2022-08-19

**Authors:** Piotr Merks, Jameason D. Cameron, Marcin Balcerzak, Urszula Religioni, Damian Świeczkowski, Mikołaj Konstanty, Dagmara Hering, Filip M. Szymański, Milosz Jaguszewski, Régis Vaillancourt

**Affiliations:** 1 Department of Pharmacology and Clinical Pharmacology, Faculty of Medicine, Collegium Medicum, Cardinal Stefan Wyszyński University, Wóycickiego 1/3, 01-938 Warsaw, Poland; 2 Children’s Hospital of Eastern Ontario, Ottawa, ON Canada; 3 Medink.eu, Warsaw, Poland; 4 School of Public Health, Centre of Postgraduate Medical Education of Warsaw, 01-826 Warsaw, Poland; 5 First Department of Cardiology, Medical University of Gdansk, 7 Dębinki Street, 80-952 Gdańsk, Poland; 6 Silesian Pharmacy Chamber, Katowice, Poland

**Keywords:** Dabigatran, Atrial fibrillation, Venous thromboembolism, Pictogram, Medication adherence, Pharmacist

## Abstract

**Backround:**

Dabigatran is a direct thrombin inhibitor used to treat cardiac arrhythmias, and rates of non-adherence to dabigatran in Polish populations are high. The current study examined how a pharmacist-led intervention of counselling with pictogram-enhanced medication instructions, and smartphone medication reminders, can improve adherence to dabigatran.

**Methods:**

A 3-month pharmacist-led intervention was conducted in community pharmacies in Poland on 325 men and women filling a dabigatran prescription for the first time. Participating pharmacies were assigned into the Control Group (*n* = 172 patients) or the Intervention Group (*n* = 153 patients). The primary outcome of this prospective study was self-reported medication adherence assessed at 3 time points (day 7, day 21, and day 90) after initiation of dabigatran.

**Results:**

Patients in the Intervention Group were significantly more adherent (mean days on Dabigatan/week) than the Control Group at 7 days (6.0 ± 0.9 vs 5.4 ± 1.1, *p* < 0.0001), 21 days (5.6 ± 1.0 vs 4.9 ± 1.3, *p* < 0.0001), and 90 days (5.5 ± 1.3 vs 4.4 ± 2.0, *p* < 0.0001), respectively. The percentage of patients in the Intervention Group who reported taking dabigatran twice/day as prescribed was significantly higher than the Control Group at 7 days (82.7% vs 71.4%, *p* = 0.0311), at 21 days (84.4% vs 58%, *p* < 0.0001), and at 90 days (78.4% vs 39.7%, *p* < 0.0001), respectively. The proportion of patients fully adherent (every day, twice/day) at 90 days was significantly higher in the Intervention Group than in the Control Group (26.1% vs 13.2%, *p* = 0.0145).

**Conclusions:**

Our findings support the role for interventions in community pharmacies in Poland to improve medication adherence, thus providing evidence for the efficacy of a pharmacist-led pictogram and smartphone-based program to support optimal dabigatran treatment.

## Background

Anticoagulation therapy is the cornerstone of treatment of several common medical conditions, including atrial fibrillation (AF) and venous thromboembolism (VTE). The lifetime risk of developing AF is estimated to be 23.8% for men and 22.2% for women [1]. People with non-valvular AF are five times more likely to suffer a stroke [2] , and one in five strokes is caused by non-valvular AF. VTE includes deep vein thrombosis and pulmonary embolism and is the third most common cardiovascular disease in Europe [3].

In Poland, the estimated number of people experiencing symptomatic deep vein thrombosis each year is 56,000 and approximately 35,000 experience pulmonary embolism [4]. For primary prevention of thromboembolic events in patients with non-valvular AF and secondary prevention in those with VTE, oral anticoagulant therapy is recommended. Until 2010, vitamin K antagonists, such as warfarin, were the only oral anticoagulants on the market in Poland [5]. Non-vitamin K antagonist oral anticoagulants (NOACs) such as dabigatran are now the preferred treatment for AF and are recommended by the European Society of Cardiology [6].

Careful adherence to NOACs is particularly important because their anticoagulant effect diminishes between 12 to 24 h after intake [6]. Lack of adherence to NOACs seriously reduces their efficacy and lower adherence to dabigatran is associated with a higher risk of mortality [7, 8] and stroke [7–9]. What is troubling is the recent findings on adherence from Polish data that indicated only 50% of the patients assessed had a plasma concentration of dabigatran within the optimal range [10]. Others have reported rates of non-adherence to dabigatran ranging from approximately 28% [7, 8] to 43% [11]. Thus, it is important to find effective means of encouraging adherence to dabigatran.

A recent systematic review of pharmacist-led interventions and medication adherence concluded that the most effective interventions were multifaceted, targeted, and personalized [12]. There is also evidence that patient education using pictograms i.e. symbols or drawings representing a specific concept, can not only make complicated information more attractive, 2006) [13], but these images can also improve comprehension and recall of proper medication-taking behavior [14–16]. As far as the authors are aware, there are currently no Polish studies examining how a multifaceted and targeted pharmacist-led intervention using the British National Health Service New Medicines Service (NMS) ( R.A., E. et al*.* Supporting adherence for people starting a new medication for a long-term condition through community pharmacies: A pragmatic randomised controlled trial of the New Medicine Service. *BMJ Qual. Saf.* 25, 747–758 (2016).) model may improve medication adherence to dabigatran. The NMS is designed to support adherence to a new medication for patients with a chronic condition because it was observed that a significant proportion of patients newly prescribed a chronic medication quickly become nonadherent.

The aim of the current intervention was to prospectively examine group differences in medication adherence in patients prescribed dabigatran for the first time. interinterviewingWe predicted that those in the Intervention Group would demonstrate higher levels of adherence relative to the Control Group.

## Design and methods

The Control Group experienced standard practice in the delivery of medication information, whereas the Intervention Group experienced standard practice plus education delivered by pharmacist counselling based on motivational interviewing principals, pictogram-enhanced medication instructions, and a smartphone that incorporated medication reminders. The primary outcome of this prospective cluster-randomized study was self-reported medication adherence assessed at 3 time points (day 7, day 21, and day 90) after initiation of Dabigatran, measured by the number of days of missed doses and the number of doses used per day, which were combined to assess full adherence to Dabigatran.

### Participants

Pharmacists were eligible to participate if they were willing to perform all training activities required and to conduct all study interventions and assessments in accordance with the study protocol. Prior to initiating recruitment, the research team verified via phone calls that the pharmacists who were at intervention locations clearly understood how to employ the modified NMS intervention and study protocol.

The initial contact with the patients was through the participating pharmacies, where each patient was screened for eligibility via a semi-structured interview performed by the pharmacist. If the patient met eligibility criteria and consented to participate, the pharmacist then administered study questionnaires and conducted all patient follow-up.

Patients from participating pharmacies were eligible if they were 18 years of age or older and had a diagnosis of non-valvular atrial fibrillation or venous thromboembolism and were filling a prescription for dabigatran for the first time. Patient participation also required that the patient consented to be contacted by phone and demonstrated sufficient Polish language skills to complete assessments (as determined by the pharmacist).

### Design and experimental procedure

Pharmacies were first recruited via a website (www.pilotazopiekifarmaceutycznej.pl). Upon responding to the study website and indicating interest to be a participating pharmacy (*e.g.* understanding the study protocol), all study documents and agreements were electronically sent whereby each pharmacy that consented to participate completed and signed all agreements and completed online testing and training to ensure compliance to the study protocol. Once the research team determined that a pharmacy was eligible to participate and the intervention pharmacists clearly understood the modified NMS tools that consisted of motivational interviewing, the pharmacy was then allocated to either the Control Group or the Intervention Group.

### Allocation of pharmacies

Pharmacies were divided into two blocks (groups), large and small, based on number of patients receiving prescription filling services at that pharmacy. Within these two blocks, pharmacies were randomized in a 1:1 ratio to intervention or control using a random number generator. Knowledge of this blocking factor is not likely to affect pharmacist behaviour during the trial in any way that could bias results.

Allocation concealment mechanism: It is not considered necessary or feasible to conceal allocation of pharmacies to intervention or control conditions. This inability to conceal the allocation is not expected to bias the results of this trial. Pharmacists will be informed of whether they are allocated to the intervention or control condition after all participating pharmacists have been enrolled.

The random allocation sequence was generated by the research team, who enrolled pharmacist participants, and assigned them to either the intervention or control condition. The pharmacists then enrolled patient participants, who were automatically be assigned to the condition to which the pharmacist was assigned.

The study took place in community pharmacies in Poland, conducted between January, 2019 and May, 2020. All participants provided informed consent, and the current study followed the ethical principles outlined in the declaration of Helsinki and was approved by the Ethical Committee in Bydgoszcz, approval number KB 463/2019.

### Control and intervention groups

Participants in the control group experienced treatment as usual in their pharmacy, where the pharmacist dispensed the medication and offered any information he or she would typically provide upon first filling a prescription for dabigatran. Participants received phone calls at 7, 21, and 90 days after filling their initial prescription. During these phone calls, the pharmacist asked the patient to answer a self-reported measure of medication adherence and the patients were free to ask any questions they had during any of their contacts with the pharmacist. The Control Group participants were free to use any reminder system, including smartphone applications similar to the one prescribed to the Intervention Group.

For the Intervention Group, upon dispensing dabigatran the pharmacist spent time with the patient in a process that involved medication reconciliation, patient education, and how to manage side effects (pharmacist education materials delivered separately). During each of phone calls, the pharmacist again employed the NMS format to enquire about barriers to treatment adherence and to help patients problem-solve concerning their adherence. In addition, the patient was provided with a pictogram-enhanced information leaflet on dabigatran written in easy to understand language and pictogram-enhanced medication labels. Each participant in the Intervention Group was also provided with a smartphone application that cued up daily medication reminders.

### Measurements

Patient demographics (age, sex, smoking status, etc.) were assessed at baseline by the pharmacist. Medication adherence was assessed via self-report from the patient when the pharmacist called on days 7, 21, and 90. The questions posed to the patients were the following: 1, “Think about the last 7 days—how many days have you been taking dabigatran?” 2. “How often during the day have you taken dabigatran in the last 7 days?” and 3. “Think about the last 7 days, what per cent of the time were you able to take all your dabigatran as your doctor prescribed it?”.

### Data analysis

Patients’ characteristics were summarised using the means and standard deviation (SD) for continuous variables and counts and percentages for categorical variables. To compare categorical variables between groups odds ratios with 95% confidence intervals (CI), the z-statistic and associated P-value were calculated. The Mann–Whitney U test was used to compare a difference in continuous variables between two groups.

## Results

### Participant flow and baseline characteristics

**Fig. 1:**
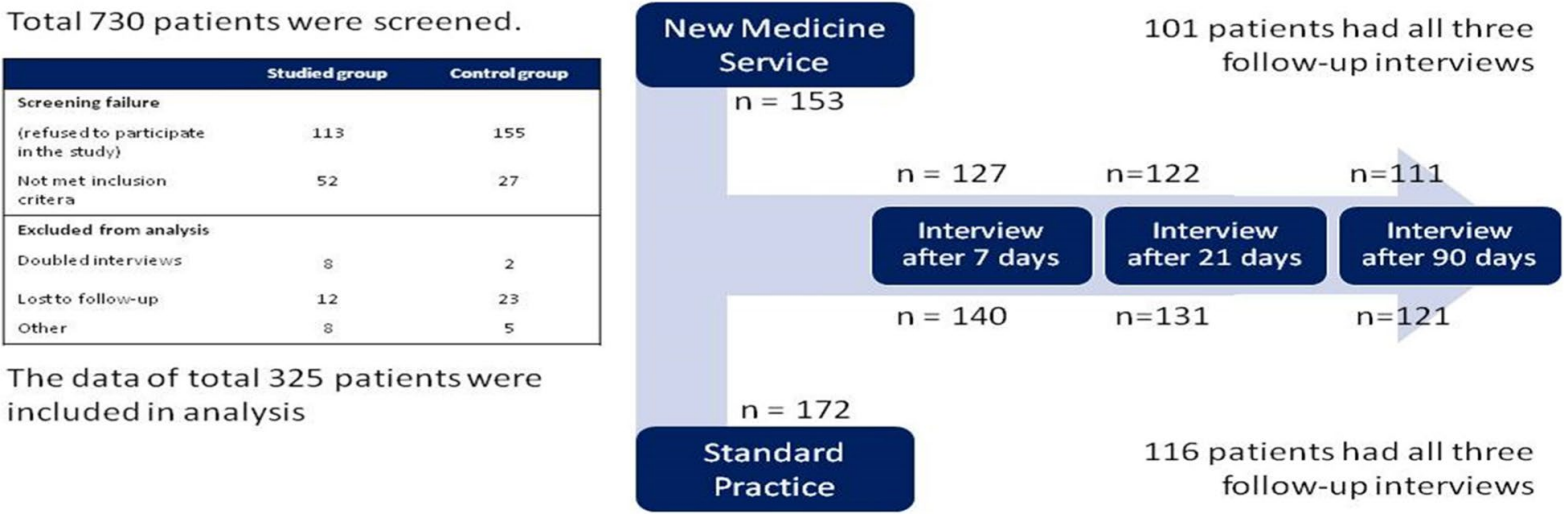
Flow Diagram of Patient Enrollment and Final Completion

A total of 730 participants were screened for study participation and 325 were randomized into two groups: Control Group (*n* = 172) and Intervention Group (*n* = 153) (see Fig. 1). At randomization, the Control Group consisted of 153 patients (90 male; 63 female) aged 67.1 ± 10.1 years. The Intervention Group consisted of 172 patients (73 male; 99 female) aged 67.6 ± 10.5 years. There was no significant difference between the groups in age (*p* = 0.695), but there were significantly more females in the Control Group (*p* = 0.003).

### Adherence to prescribed dose (twice/day)

**Fig. 2:**
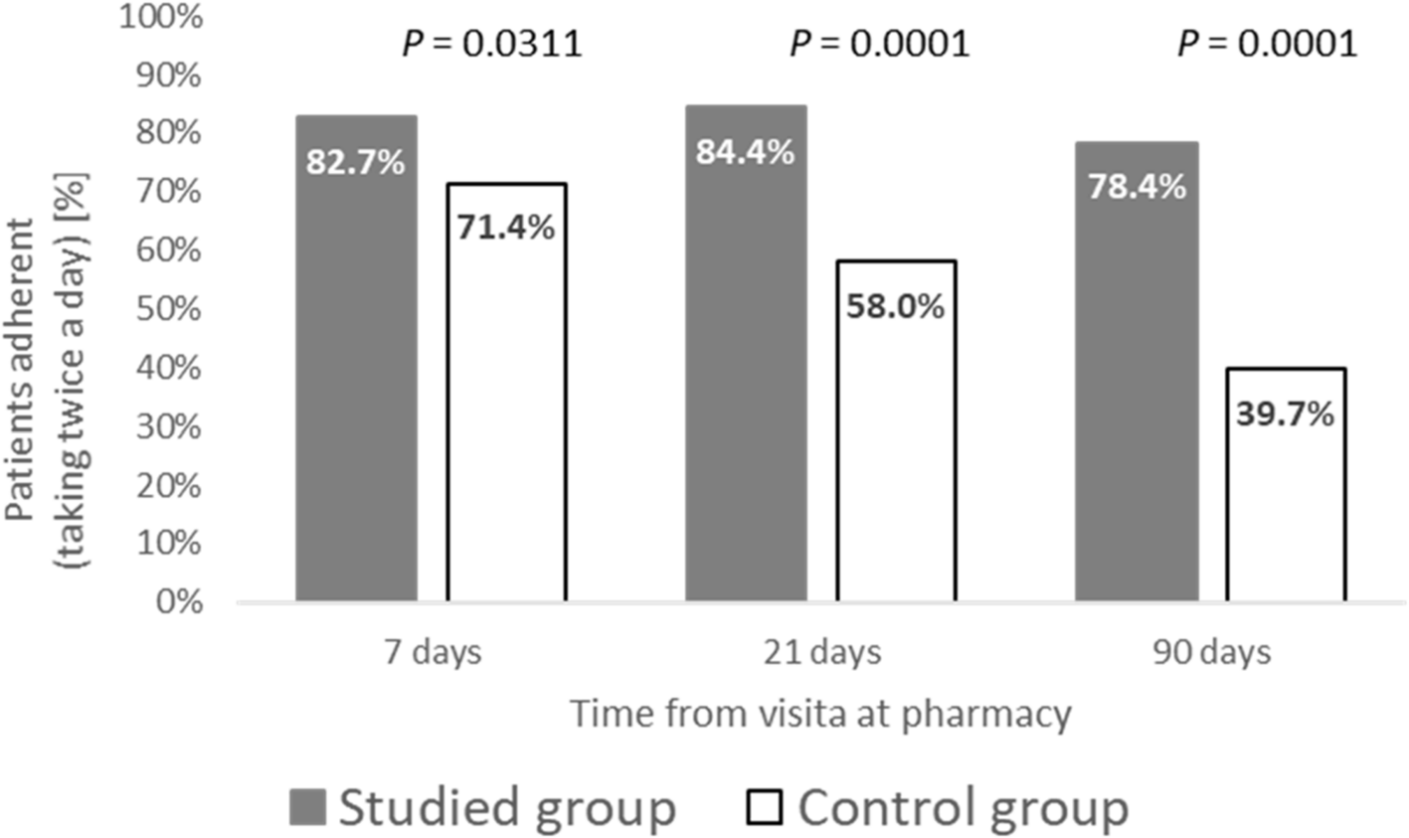
Adherence to the Prescribed Dose of Dabigatran (twice/day) by Group at 7, 21, and 90 Days After Initiating Treatment (Studied group = Intervention group)

The percentage of patients in the Intervention Group who reported taking dabigatran twice/day as prescribed was significantly higher than the Control Group at 7 days (82.7% of patients vs. 71.4%; OR 1.91, 95% CI: 1.06–3.44, *p* = 0.031), at 21 days (84.4% vs. 58%; OR 3.92, 95% CI: 2.15–7.15, *p* < 0.0001), and at 90 days (78.4% vs. 39.7%; OR 5.51,95% CI: 3.08–9. 85, *p* < 0.0001), respectively (Fig. 2).

### Adherence to daily usage (days/week)

**Fig. 3:**
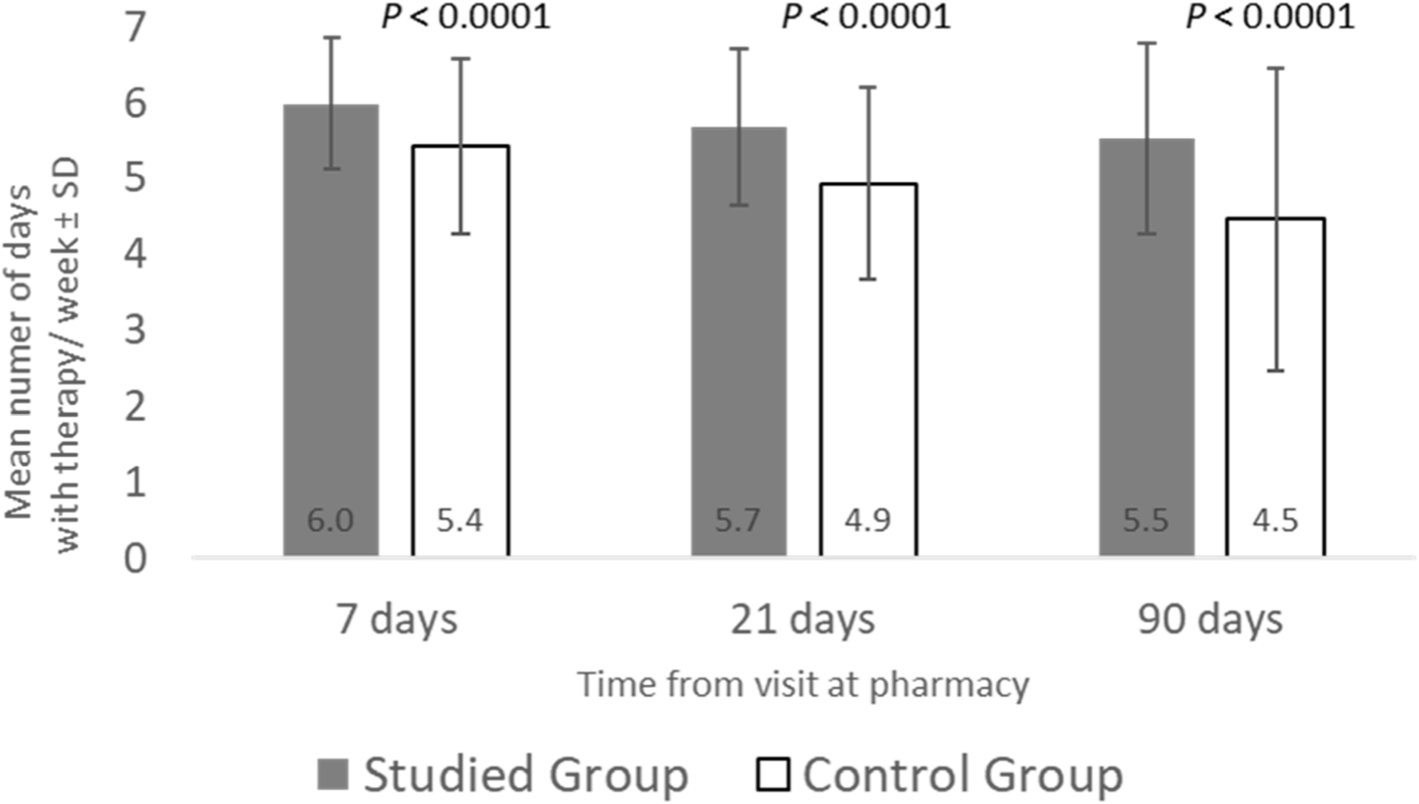
Mean Number of Days per Week on Dabigatran by Group at 7, 21, and 90 Days After Initiating Treatment (Studied group = Intervention group)

Patients in the Intervention Group were significantly more adherent (mean days on dabigatan/week) than the Control Group at 7 days (6,0 ± 0.9 vs 5.4 ± 1.1, *p* < 0.0001), 21 days (5.6 ± 1.0 vs 4.9 ± 1.3, *p* < 0.0001), and 90 days (5.5 ± 1.3 vs 4.4 ± 2.0, *p* < 0.0001), respectively (see Fig. 3). In both groups, a median number of days on treatment/ week decreased over time. However, the decline in adherence to standard treatment from day 7 to day 90 was lower in the Intervention Group (0.5 days/week, *p* = 0.006) compared to the Control Group (1 day/week, *p* = 0.0001) (results not shown).

### Fully adherent patients (every day, twice/day)

**Fig. 4:**
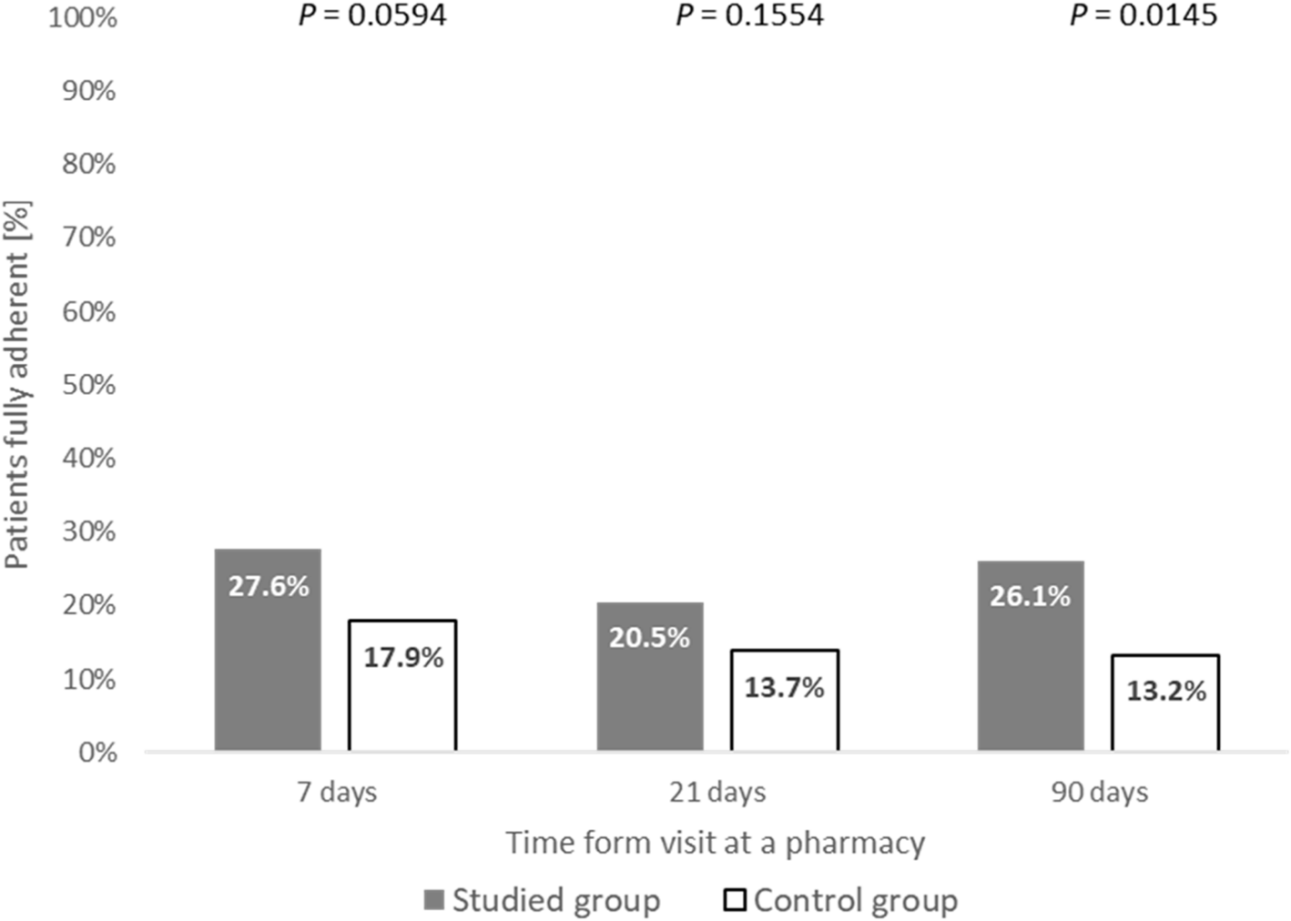
Proportion of Patients Fully Adherent (every day, twice per day) to Dabigatran Treatment by Group at 7, 21, and 90 Days After Initiating Treatment (Studied group = Intervention group)

The proportion of patients fully adherent (every day, twice/day) at 90 days was significantly higher in the Intervention Group than in the Control Group (26.1% vs 13.2%; OR = 2.32, 95% CI 1.18 – 4.56, *p* = 0.0145)**.** There was no significant difference in fully adherent patients by the group at Day 7 or Day 21 (*p* > 0.05) (see Fig. 4).

## Discussion

Dabigatran is a direct thrombin inhibitor used to treat cardiac arrhythmias, and rates of adherence to dabigatran in Polish populations are low. It is unclear if low rates of adherence are due to the fact that standard pharmacist care in Poland is typically limited to the dispensing of medications with little advice about dosage or how to store the medication [17]. The current intervention examined how pharmacist counselling based on motivational interviewing principles, pictogram-enhanced medication instructions, and smartphone medication reminders can improve adherence to dabigatran compared to standard pharmacy practice in Poland. Across all time points, there was a significantly higher proportion of patients in the Intervention Group who did not miss a daily dose (*p* < 0.05) and who were adherent to the prescribed dose (twice/day) (*p* < 0.0001) when compared to the Control Group. Furthermore, the proportion of patients fully adherent (every day, twice/day) at the end of the study was significantly higher in the Intervention Group (*p* < 0.05)**.**

Research suggests that there is often a precipitous decline in adherence to medications in chronic patients over time [18–20], and it is known that low adherence to dabigatran is associated with a higher risk of complications, including combined all-cause mortality and stroke [8]. The fact that the Intervention Group had a higher proportion of patients using the drug twice daily as prescribed suggests that our pharmacist-led intervention increased awareness about proper dosing of dabigatran. It is important to note that we saw group differences suggesting positive intervention effects after 7 days (14% increase in adherence), after 21 days (31% increase in adherence), and after 90 days (50% increase in adherence). The difference between groups was statistically significant at all time points (see Fig. 2).

Common intervention approaches such as the British National Health Service New Medicines Service (NMS) [21] have been developed as a framework to improve adherence, and the current intervention was modelled similarly, including ongoing education and support. In the current study, the Intervention Group showed a 2% increase in adherence to the prescribed daily dose from day 7 to day 21 and only a 5% decrease in adherence from day 7 to day 90. This is in stark contrast with the Control Group, whose adherence to the prescribed daily dose decreased ~ 19% from day 7 to day 21, and dropped ~ 45% from day 7 to day 90. Indeed, a recent systematic review on pharmacist-led interventions and adherence has found that pharmacists can reduce barriers to adherence and improve disease control by combining NMS elements such as patient education, follow-up, and monitoring [12].

Examining the number of days per week that patients were adherent to dabigatran, patients in the Intervention Group were significantly more adherent across all time points (see Fig. 3). Relative to the Control Group, the improvements in weekly adherence were noted in the first week, and importantly, after 90 days only for those in the Intervention Group. Specifically, although the median number of days on treatment per week decreased over time for both groups, the decline in adherence to everyday treatment from day 7 to day 90 was lower in the Intervention Group (0.5 days/week, *p* = 0.006) compared to the Control Group (1 day/week, *p* = 0.0001). This increased compliance noted in the Intervention Group is likely to have clinical relevance and is particularly important because the anticoagulant effect of dabigatran diminishes between 12 to 24 h after intake [6].

The proportion of patients fully adherent (every day, twice/day) did not differ by the group until the end of the study (see Fig. 4). At study completion, the Intervention Group demonstrated nearly 50% more patients who were fully adherent to treatment. Our findings are encouraging in the current context of dabigatran treatment in Poland, where nearly 50% of the patients assessed had a plasma concentration of dabigatran outside of the optimal range [10]. Taken together, and with the understanding that medication adherence is best promoted with the aid of health professionals such as pharmacists [22], our findings highlight that interventions that focus on dose simplification—with ongoing tailored support—are successful at improving patient outcomes for adherence. Furthermore, due to the limited availability of one-on-one counselling in standard pharmacy practice in Poland, efforts should be made to identify “high-risk” patients with an easily calculated CHADS_2_ Score [23].

In modern healthcare environments, physicians and pharmacists tend to have less time to spend on education for the patient [22], so there is a need to focus on the relationship between self-management or self-efficacy from the patient, and an open dialogue with the healthcare team. In practice, patients are often sent home with a set of simplified medication instructions on information leaflets, supplementing verbal counselling with written instructions [24, 25]. There is a good evidence that educational leaflets with accurate, straightforward and comprehensible wording, as well as picture-based instructions, are good ways to support verbal medical advice [26, 27]. The guiding theory of pictogram use in any field of study is that when exposed to an image, the verbal memory may be triggered, reinforcing memory traces and subsequent recall. In order to do so, the message needs to be clear, appropriate for the intended audience and must focus on actions rather than information [28, 29]. Although the current study was not designed to assess the independent effects of the various elements of our intervention (e.g. counselling, pictograms, smartphone alerts, etc.), it showed that a pharmacist-led intervention can improve adherence to dabigatran in Poland.

Limitations of the study include the self-reporting of medication adherence by patients. In order to maintain external validity, the Control Group was allowed to use their own smartphone reminder system, and it is unclear if this could have impacted how adherence differed between groups. Since the intervention consisted of several elements, similar to other medication adherence interventions, it is difficult to ascertain which component of the intervention contributed to the improvement in medication-taking behaviour. The decision to focus on patients filling the first prescription is a strength of the study as it provides an accurate baseline for medication adherence and it has been estimated that nearly half of patients starting a new medication for chronic disease will become non-adherent within the first six months of treatment. Despite not having in-depth training in motivational interviewing, the data still showed that patients in the intervention group demonstrated improved compliance. The current study design increases the external validity of the findings, thus making our approach easy to implement in standard clinical practice. Despite the fact that the quality of the pharmacist counselling was not assessed as part of the study design, the current findings still demonstrated that the current intervention can significantly improve patient compliance to medication.

## Conclusions

Their regular patient contact provides community pharmacists with many opportunities to identify and follow up on patients’ adherence issues. Across all time points in this pharmacist-led intervention, there was a significantly higher proportion of patients in the Intervention Group who did not miss a daily dose and were adherent to the prescribed dose (twice/day) when compared to Controls. At study completion, the proportion of patients fully adherent to dabigatran treatment was significantly higher in the Intervention Group, thus providing evidence for the efficacy of an NMS-modelled program to improve medication adherence in patients initiating dabigatran treatment in Poland. Our findings support the role of a pharmacist-led intervention to improve medication adherence and that effective spoken and written communication of information about medicine, in combination with health-related pictograms, can lead to improved treatment outcomes for patients taking dabigatran.

## Data Availability

All data are available from the corresponding author (dr Urszula Religioni).

## Abbreviations

AF: Atrial fibrillation
VTE: Venous thromboembolism
NOACs: Non-vitamin K antagonist oral anticoagulants
